# Kaiso mediates human ICR1 methylation maintenance and *H19* transcriptional fine regulation

**DOI:** 10.1186/s13148-016-0215-4

**Published:** 2016-05-04

**Authors:** Florian Bohne, David Langer, Ursula Martiné, Claudia S. Eider, Regina Cencic, Matthias Begemann, Miriam Elbracht, Luzie Bülow, Thomas Eggermann, Ulrich Zechner, Jerry Pelletier, Bernhard Ulrich Zabel, Thorsten Enklaar, Dirk Prawitt

**Affiliations:** Centre for Paediatrics and Adolescent Medicine, University Medical Centre, Langenbeckstr. 1, 55101 Mainz, Germany; Department of Biochemistry and the Rosalind and Morris Goodman Cancer Research Centre, McGill University, Montreal, Quebec H3G 1Y6 Canada; Institute of Human Genetics, RWTH Aachen University, Pauwelsstr. 30, 52074 Aachen, Germany; Institute of Human Genetics, University Medical Centre, Langenbeckstr. 1, 55101 Mainz, Germany; Centre for Paediatrics and Adolescent Medicine, University Medical Centre, Mathildenstr. 1, 79106 Freiburg, Germany; Centre for Paediatrics and Adolescent Medicine, University Medical Centre, Obere Zahlbacher Str. 63, 55131 Mainz, Germany

## Abstract

**Background:**

Genomic imprinting evolved in a common ancestor to marsupials and eutherian mammals and ensured the transcription of developmentally important genes from defined parental alleles. The regulation of imprinted genes is often mediated by differentially methylated imprinting control regions (ICRs) that are bound by different proteins in an allele-specific manner, thus forming unique chromatin loops regulating enhancer-promoter interactions. Factors that maintain the allele-specific methylation therefore are essential for the proper transcriptional regulation of imprinted genes. Binding of CCCTC-binding factor (CTCF) to the IGF2/H19-ICR1 is thought to be the key regulator of maternal ICR1 function. Disturbances of the allele-specific CTCF binding are causative for imprinting disorders like the Silver-Russell syndrome (SRS) or the Beckwith-Wiedemann syndrome (BWS), the latter one being associated with a dramatically increased risk to develop nephroblastomas.

**Methods:**

Kaiso binding to the human ICR1 was detected and analyzed by chromatin immunoprecipitation (ChIP) and electrophoretic mobility shift assays (EMSA). The role of Kaiso-ICR1 binding on DNA methylation was tested by lentiviral Kaiso knockdown and CRISPR/Cas9 mediated editing of a Kaiso binding site.

**Results:**

We find that another protein, Kaiso (ZBTB33), characterized as binding to methylated CpG repeats as well as to unmethylated consensus sequences, specifically binds to the human ICR1 and its unmethylated Kaiso binding site (KBS) within the ICR1. Depletion of Kaiso transcription as well as deletion of the ICR1-KBS by CRISPR/Cas9 genome editing results in reduced methylation of the paternal ICR1. Additionally, Kaiso affects transcription of the lncRNA *H19* and specifies a role for ICR1 in the transcriptional regulation of this imprinted gene.

**Conclusions:**

Kaiso binding to unmethylated KBS in the human ICR1 is necessary for ICR1 methylation maintenance and affects transcription rates of the lncRNA *H19*.

**Electronic supplementary material:**

The online version of this article (doi:10.1186/s13148-016-0215-4) contains supplementary material, which is available to authorized users.

## Background

**Fig. 1:**
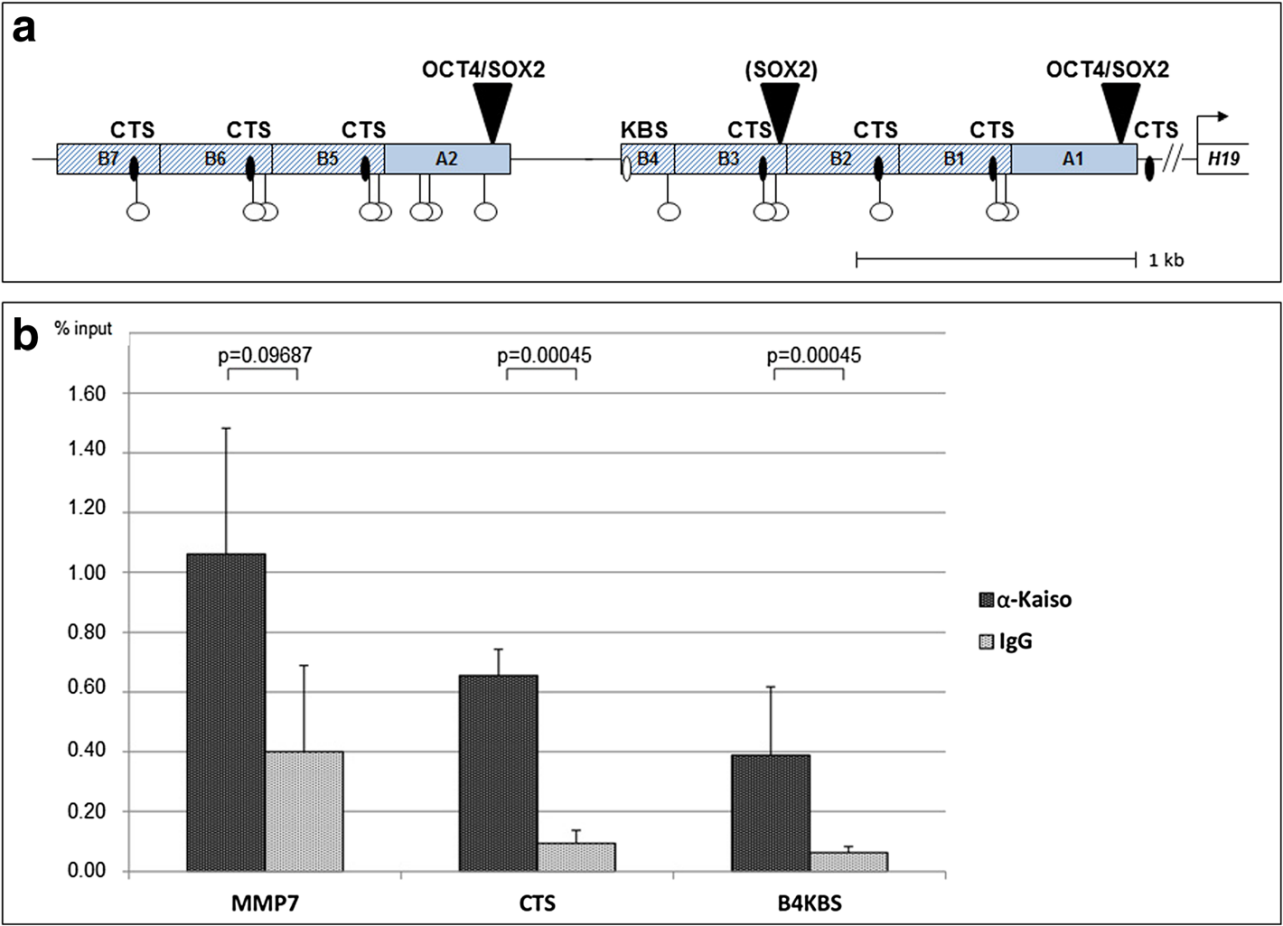
Human ICR1 is bound by Kaiso. **a** Schematic overview of the maternal human ICR1 consisting of two clusters each containing one 450-bp (A-) repeat with OCT4/SOX2 binding sites, followed by three to four 400-bp (B-) repeats. Each B-repeat, with the exception of the shortened B4-repeat, contains a CTS (*black ovals*) and a ZFP57 binding site. An optimal KBS (5′TNGCAGGA3′) resides in B4 (*white oval*). KBS-similar motifs (5′TNGCAGGC3′) can be detected in repeats B1, B2, B3, B5 and B6. *White lollipops* indicate CGCG motives as putative binding sites for Kaiso when methylated. **b** Chromatin immunoprecipitation (ChIP) with a specific anti-Kaiso antibody from primary fibroblast cell culture extracts indicates precipitation of the B-repeat DNA (CTS containing repeats B1, B2, B3, B5 and B6) as well as ICR1-B4-DNA (B4-KBS). Binding of Kaiso to the known functional KBS in the MMP7 promoter was used as a positive control for the ChIP. Data are represented as mean ± SEM from four independent ChIP assays, and enrichment was normalized against the input. Relative DNA concentrations were analysed by qPCR indicating significant pulldowns for MMP7, CTS and B4-KBS. *P* values for the individual pulldowns are indicated

Imprinting disorders (IDs) are rare congenital diseases and arise from the dysregulation or mutation of imprinted genes. Causative imprinted genes are clustered in groups and regulated by the so-called imprinting control regions (ICRs) that are differentially methylated (reviewed in [1]). The human imprinting control region 1 (ICR1) located on chromosome 11p15.5 regulates the allele-specific expression of the *IGF2* and *H19* genes. It contains two different types of repetitive sequences (2 A- and 7-B-repeats) with seven CTCF binding sites, six of which are located in B1, B2, B3, B5, B6 and B7. The seventh CTCF binding site is located downstream from A1. As well, in ICR1, A1 and A2 are two OCT4 and two SOX2 binding sites [2]. Differential parental methylation has been proposed for all B-repeats harbouring CTCF target sites (CTS) in most normal tissues [3–6] (see Fig. 1). The growth disorders Beckwith-Wiedemann syndrome and Silver-Russell syndrome are linked to opposite DNA methylation defects involving the ICR1. OCT4 and SOX2 bind their target sites on the unmethylated maternal allele and prevent a gain of methylation on this allele. Consequently, CTCF binds to the CTS on the unmethylated maternal ICR1 and creates a functional chromatin boundary thus blocking the interaction between shared enhancer elements with the *IGF2* promoters [7]. Studies in murine models [8–11] as well as in human cells [12] have shown that the higher-order chromatin structure of the maternal and paternal *Igf2/H19* domains differ and form characteristic loops that confer the described effects and indicate CTCF to be the master regulator of ICR1 function. On the methylated paternal allele, CTCF binding is prevented due to the methylation of the recognition site, triggering *IGF2* expression from the paternal allele by permitting the promoter enhancer interactions [13–15]. This methylation of the CTS in the ICR1 is established during late paternal gametogenesis and maintained after fertilization of the oocyte and in the somatic cells. Methylation of ICR1 generates binding sites for the zinc finger protein ZFP57 in all B-repeats. In other differentially methylated regions (DMR), like KvDMR1/ICR2 in 11p15.5 or SNRPN in 15q11.2, binding of ZFP57/KAP1 to the methylated target site leads to a ZFP57-dependent methylation maintenance in ES cells [16]. However, methylation maintenance in the ICR1 (and *Igf2r*-DMR) has been described to be independent of ZFP57 binding [17]. Another protein that may contribute to the methylated ICR1-mediated effects is ZBTB33 (Kaiso), a member of the pox virus and zinc finger (POZ) family of zinc finger (ZF) transcription factors that can bind methylated C^m^GC^m^G motifs via its three Krüppel-like C_2_H_2_ ZFs. In the presence of an appropriate consensus sequence around a single CG dinucleotide, it also recognizes the C^m^G motif [18]. Kaiso is known to associate with p120 catenin and is a constituent of one of two methyl-CpG binding complexes originally designated as MeCP1 [19]. It behaves as a methylation-dependent transcriptional repressor. Filion and coworkers performed chromatin immunoprecipitation (ChIP) with antibodies directed to Kaiso on murine brain extracts and showed binding of Kaiso mostly, but not exclusively to the paternal (methylated) ICR1 [20]. Kaiso is the only POZ-ZF protein known to be able to bind methylated CG motifs [18, 19], as well as the specific DNA sequence ^5′^TNGCAGGA^3′^ (Kaiso binding site (KBS)) with even higher affinity [21]. It is unclear whether binding of Kaiso to the KBS or methylated targets represent separate activities of Kaiso. Binding of Kaiso to the KBS seems to be evolutionarily linked to the appearance of genomic imprinting since only the methyl-CpG binding function of Kaiso is evolutionarily conserved in frog, fish and chicken [22], suggesting a distinct role for KBS in mammalian organisms. Therefore, we examined the human ICR1 for the presence of consensus unmethylated Kaiso binding sites [21] and uncovered a unique KBS (^5′^TGGCAGGA^3′^) in the B4-repeat, which most interestingly has no CTS (see Fig. 1a). Additionally, KBS cognate motifs (^5′^TNGCAGG**C**^3′^) exist in B1, B2, B3, B5 and B6. The partially binding of Kaiso to the unmethylated allele [20] and the presence of a KBS, which can potentially be recognized bound by Kaiso on both alleles, led us to investigate whether Kaiso plays a role in the maintenance of human genomic imprinting via binding at the ICR1.

## Results

### Kaiso binds the ICR1 in primary human fibroblasts

To determine whether Kaiso binds the ICR1 in primary cells, we performed ChIP experiments with primary human fibroblast cells that have a paternally methylated and a maternally unmethylated ICR1 sequence. Full-length ICR1-B fragments (B1, B2, B3, B5 and B6) as well as the B4-repeat were detected in pulldowns from the anti-Kaiso antibody (Fig. 1b). Based on the presence of the CGCG motif, but not the CG motif in the optimal sequence context [18] in every single B-repeat including the B4-repeat, it is difficult to distinguish whether the unmethylated KBS in B4 is directly occupied in vivo or if the pulldown is occurring due to interactions with a neighbouring C^m^GC^m^G site (see Fig. 1a). Precipitation of the full-length B-repeats is 1.5-fold higher than the precipitation of the B4-KBS-DNA, probably reflecting the abundance of Kaiso binding within the methylated B-repeats, compared to the single B4-KBS. Binding of Kaiso to the known functional KBS in the MMP7 promoter [22] was used as a positive control for ChIP and suggests binding of Kaiso to the ICR1 comparable to the MMP7-promoter. These results indicate that Kaiso binds the human ICR1 including its KBS in the B4-repeat. While the full-length B-repeat binding is observed on the methylated paternal allele, binding of the B4-KBS can occur on both alleles, since the KBS cannot be methylated.

### Kaiso binds to unmethylated consensus sequence in the human ICR1-B4-repeat

**Fig. 2:**
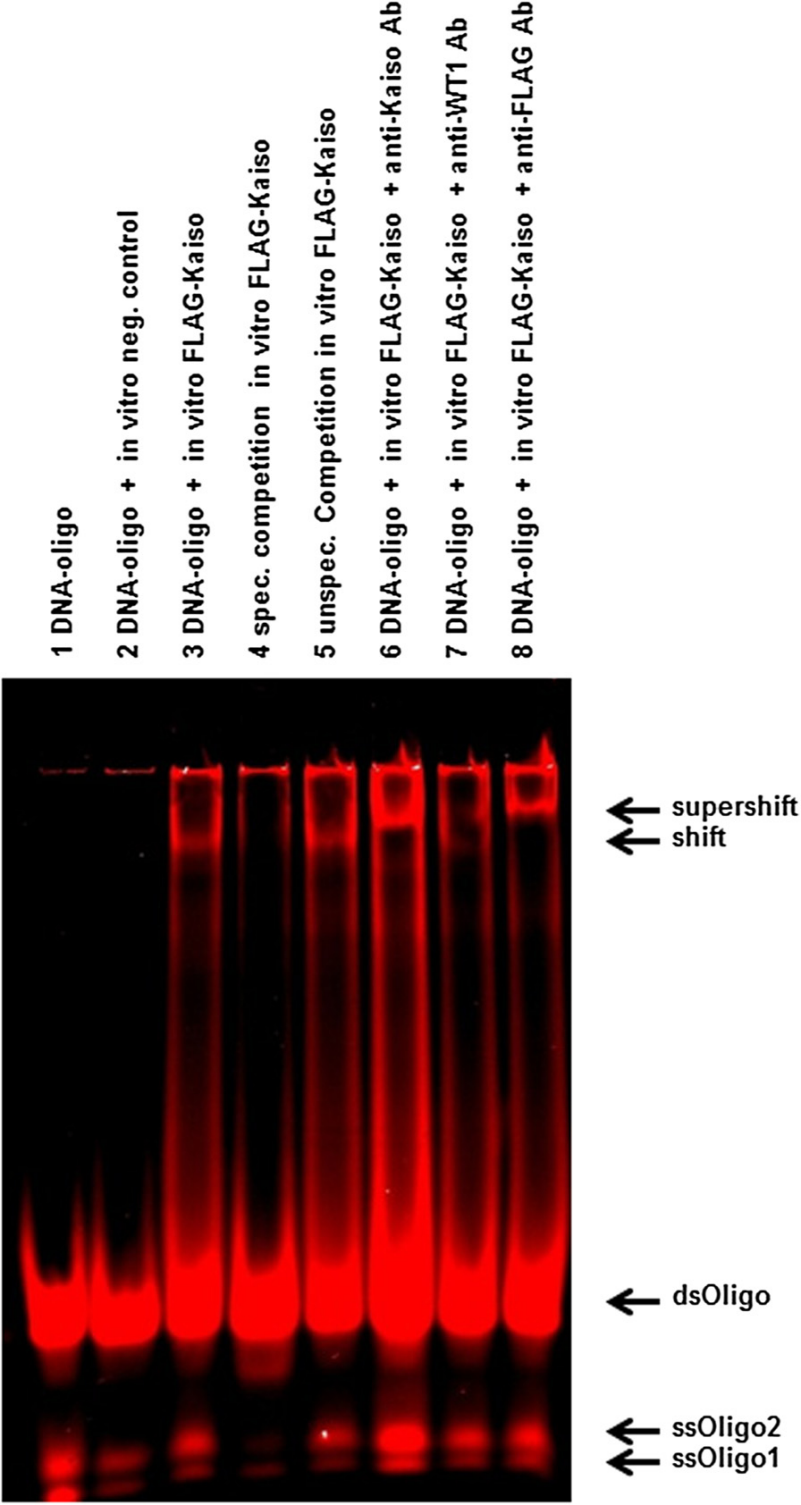
Kaiso binds the ICR1-B4-KBS in vitro. EMSA was carried out with fluorescence-labelled oligonucleotide probes for the ICR1-B4-KBS (lanes *1*–*8*). N-terminal Flag-tagged Kaiso protein (in vitro *FLAG-Kaiso*) was produced by in vitro translation, using modified HeLa cell extracts (Pierce). The ds-oligo for the ICR1-B4-KBS (lane *1*) is shifted by in vitro *FLAG-Kaiso*—Kaiso (lanes *3* and *5*), but not by the cell extract containing the expression vector alone (neg. control, lane *2*). Complexes were competed by an excess of unlabelled ICR1-B4-KBS ds-oligo (lane *4*) but not with a non-specific control oligo (lane *5*). In supershift reactions, Flag-tagged Kaiso was incubated with anti-Kaiso (lane *6*), anti-WT1 (lane *7*) or anti-Flag (lane *8*) antibodies, the anti-WT1 representing a non-specific control

In vitro binding of Kaiso to fragments of the unmethylated ICR1 was tested by EMSA using fluorescence double-stranded oligonucleotide sequences harbouring the determined putative KBS sequences. The putative KBS located in B4 [21] showed a mobility shift in the presence of Kaiso in contrast to the cognate KBS of the B1-, B2-, B3-, B5- and B6-repeats. The shift can be competed by an excess of unlabelled KBS double-stranded oligonucleotide sequences which abrogate Kaiso binding to ICR1-B4-KBS (Fig. 2). Kaiso binding specificity to the ICR1-B4-KBS was further confirmed using an anti-Kaiso antibody in a supershift assay. Control reactions with unspecific WT1—instead of Kaiso—antibody assure specificity of the supershift reactions. These results demonstrate that Kaiso binds the KBS in the B4-repeat of human ICR1 in vitro (Fig. 2).

### Kaiso binding to the human ICR1 affects ICR1 methylation and transcriptional activity of *H19* and *IGF2*

**Fig. 3:**
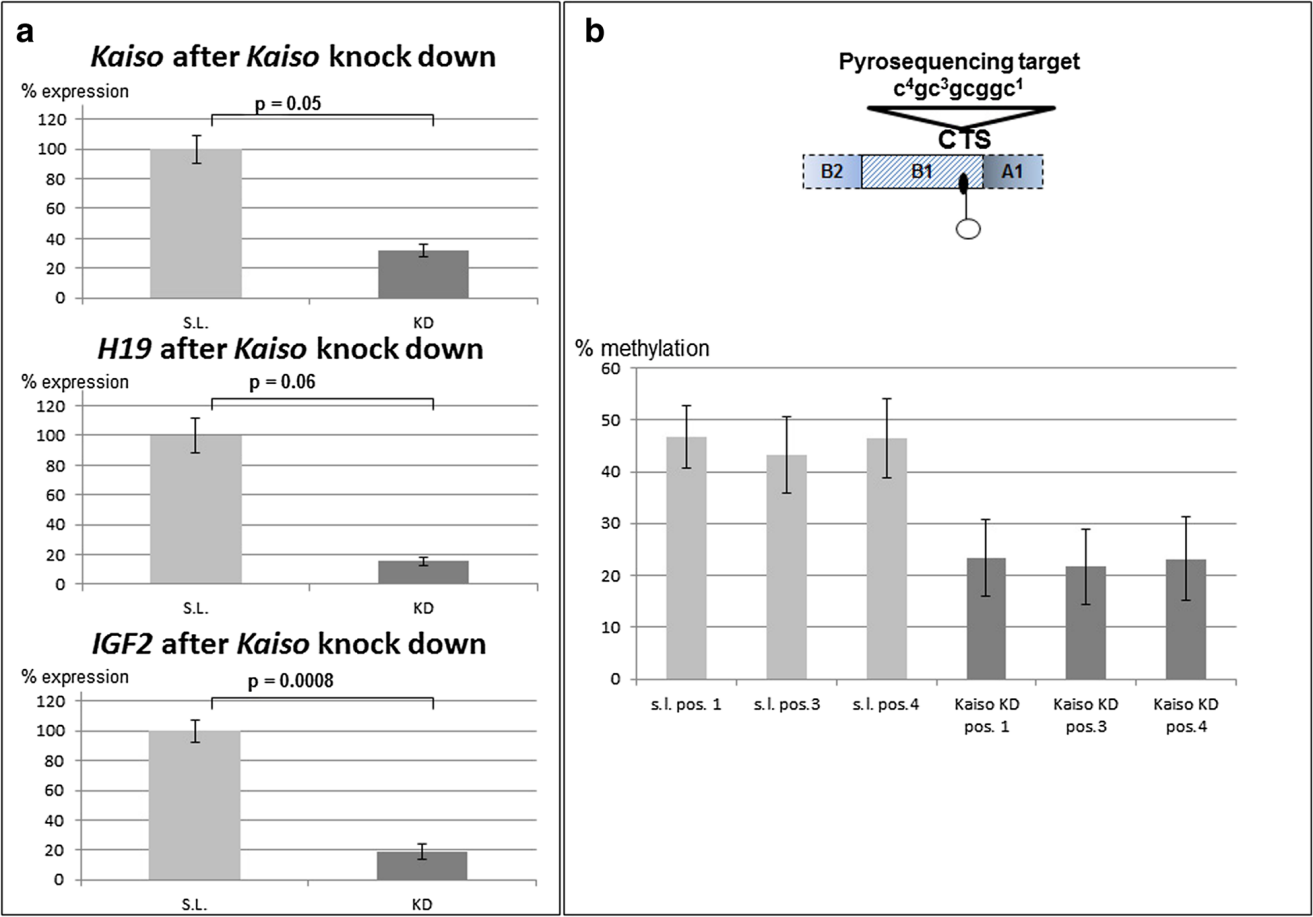
Lentiviral-mediated Kaiso *knockdown* (Kaiso *KD*) is associated with a reduction of ICR1 methylation and decreased endogenous *IGF2* and *H19* transcription in human primary. As controls, transfections with shRNA against a scrambled locus (*S.L.*) were performed. **a** Knockdown efficiency was quantitated by RT-qPCR. The expression values are normalized to *PDH* and *B2M* transcripts and are represented as mean ± SEM from two independent experiments each. *p* values for the transcriptional comparisons are indicated. **b** ICR1 methylation was determined by pyrosequencing of the three established differentially methylated ICR1 CpGs in bisulfite-treated DNA. The location of the analysed CpGs is schematically depicted on *top*. Mean methylation values of three replicates are presented ± SEM for each CG position. The knockdown of Kaiso is associated with reduced methylation of endogenous ICR1

To determine whether Kaiso binding to the ICR1 is mediating the regulatory effects of the ICR1, we examined the effect of RNAi-mediated Kaiso knockdown. We sought to chronically deplete Kaiso in primary human fibroblast cells using a lentivirus expressing a shRNA-targeting Kaiso. Following selection for stable integrants, Kaiso-shRNA-expressing fibroblast cells had only ~32 % of endogenous Kaiso mRNA (Fig. 3a) associated with a reduction of endogenous ICR1 methylation to 23 % compared to 46 % average methylation of the controls (Fig. 3b). The Kaiso knockdown did not significantly alter the differential methylation at the KvDMR1 of ~50 % (Additional file 1: Figure S1A). The reduction in ICR1 methylation in the primary fibroblasts, correlated with a significant transcriptional downregulation of *IGF2* (15 % of control) and of *H19* transcripts (8 % of control) (Fig. 3a). These data suggest a role for Kaiso in the methylation maintenance of the paternal ICR1.

### Kaiso binding to unmethylated KBS in human ICR1-B4 is necessary for ICR1 methylation maintenance

**Fig. 4:**
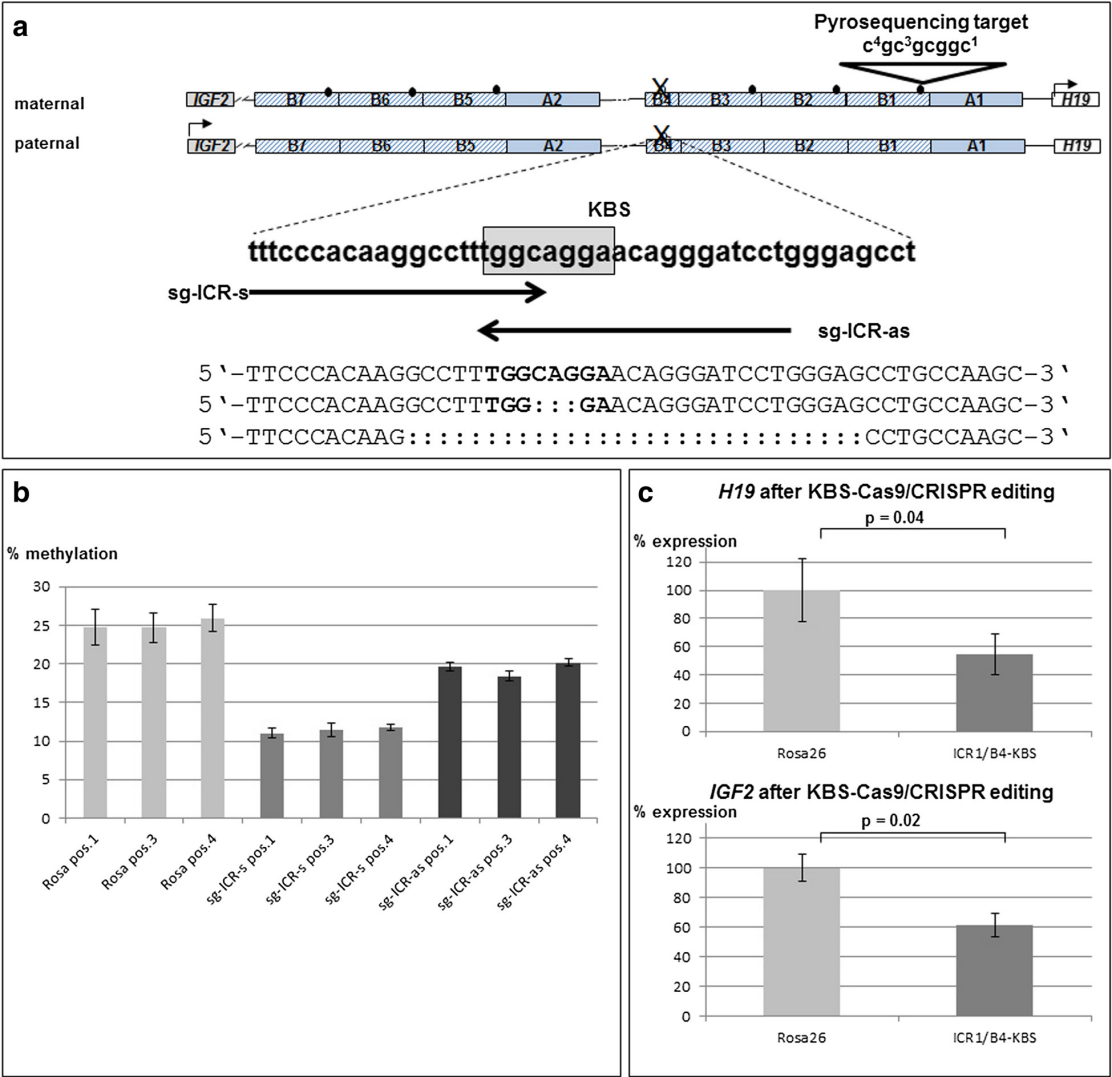
Genomic CRISPR/Cas9 deletion of the ICR1-B4-KBS leads to the loss of ICR1 methylation and transcriptional deregulation of H19 and IGF2. **a** Sense and antisense sgRNAs (sg-ICR-s, sg-ICR-as) were designed to target the KBS site. Adjacent to both sgRNAs is the presence of a PAM (^5′^NGG^3′^). After lentiviral transduction in primary fibroblasts with a differentially methylated ICR1 (50 % methylation), GFP^+^ cells were sorted by FACS. The target sequence was amplified by PCR and subcloned, and a random selection of 20 clonal integrates was sequenced. Seven out of these 20 clones showed a deletion of the target site. The smallest and largest of the detected Cas9-mediated deletions (ranging from 3 to 29 bp) are depicted here to illustrate the deletion range. All detected deletions were found to disrupt the KBS motif. **b** Cas9-modified fibroblasts are impaired for ICR1 methylation. For the following analyses, GFP^+^ cells were sorted by FACS and analysed as pooled cells. Displayed histogram values are represented as mean ± SEM from four replicate analyses for each CG position. **c** Expression of *H19* and *IGF2* is altered upon KBS editing. Expression values obtained by qRT-PCR from RNA of pooled FACS-sorted GFP^+^ cells were normalized against *PDH* and *B2M* transcripts and are shown in mean ± SEM from 2 to 3 replicate analyses each

As the observed effects of Kaiso binding on the methylation of the ICR1 could be due to binding to C^m^GC^m^G sites as well as the unmethylated KBS in the B4-repeat or even to genome-wide Kaiso depletion, we aimed to directly perturb the Kaiso binding site. Since our data suggested that a single unmethylated KBS exists in the ICR1-B4-repeat, we edited this binding site in primary fibroblasts using clustered regularly interspaced short palindromic repeats (CRISPR)/CRISPR associated 9 (Cas9) genome editing [23]. Two synthetic guide RNAs (sgRNA) targeting ICR1-B4-KBS were designed for disruption of ICR1-B4-KBS by non-homologous end joining (NHEJ) (Fig. 4a). A sgRNA targeting the murine Rosa26 locus was used as a neutral control [23]. Lentiviral transduction of fibroblasts was used to introduce Cas9, each sgRNA and a GFP reporter. GFP^+^ cells were sorted by flow cytometry and the B4-repeat was amplified by PCR and subcloned in pCDNA3. Sequencing analyses detected deletions in 7 out of 20 clones analysed (Fig. 4a). The GFP^+^ sorted cells infected with the B4-KBS targeting guide RNAs showed ~50 % reduction of ICR1 methylation compared to the ICR1 methylation observed in Rosa26-targeted fibroblasts (Fig. 4b). Like in the Kaiso knockdown approaches, the ~50 % methylation of the KvDMR1 was not significantly altered in the edited fibroblasts (Additional file 1: Figure 1B). This is consistent with the notion that binding of Kaiso to its unmethylated KBS in the B4-repeat of the ICR1 is necessary to maintain methylation on the paternal ICR1 allele. *IGF2* and *H19* transcription was significantly downregulated after the disruption of the ICR1-B4-KBS (Fig. 4c). It is also noteworthy to mention that the CRISPR/Cas-edited fibroblasts had a reduced methylation of the analysed ICR1 CpGs in both the Rosa26 controls and in the two KBS-sgRNA approaches. This might reflect a side effect of the editing machinery, although it remains unclear why it affects only the ICR1.

## Discussion

The presented results indicate that Kaiso binds to the human ICR1 and to the KBS in the ICR1-B4-repeat. Permanent downregulation of Kaiso results in a decreased methylation maintenance and a downregulation of *IGF2* and *H19* transcription. In this setting, it remains unclear if the observed effects are due to the general downregulation of Kaiso, which alters the transcription of a transfactor or reflects local effects of Kaiso binding to the methylated CGCG sequences on the paternal ICR1 and/or to the B4-KBS on both alleles. The CRISPR/Cas9 approach suggests that the methylation maintenance and the transcriptional effects are causally connected with the binding of Kaiso to the B4-KBS. Our data does not allow a determination as to whether Kaiso binds the latter on both alleles, but the observed methylation effects of Kaiso reduction and binding depletion suggest that KBS binding on the methylated paternal allele is crucial.

How can Kaiso binding mediate the methylation maintaining effects? We do not observe a complete demethylation after editing of ICR1-B4 KBS locus, which could be ascribed to incomplete editing in the pooled cells. Indeed, upon analysis of the GFP^+^ sorted primary fibroblasts, only approximately one third of the randomly analysed target sequences showed editing at the target site. In addition, since both parental ICR1 alleles can be edited and the maternal allele is unmethylated, editing at this locus will always provide a relative readout. Hence, the observed demethylation strongly suggests that Kaiso binding to the ICR1-B4-KBS is essential to maintain the methylation of the paternal ICR1 and thus affects the transcription of the imprinted genes *IGF2* and *H19*. As expected, the observed function of Kaiso did not affect the KvDMR1/ICR2 methylation, which is known to have a ZFP57-dependent methylation maintenance [16], further supporting the idea that the observed Kaiso effects are specific and represent the long-sought alternative methylation maintenance factor for ICR1 [17]. How can this function be mediated? Like in the ICR1, it has been shown for the *RB1* locus that CTCF binding protects against DNA methylation [24]. When this region gets methylated (e.g. in cancer cells), Kaiso binds to its methylated recognition sites and induces epigenetic silencing of the promoter, likely via associated repressor components that include N-CoR and HDACs [20, 24, 25]. Crystal analyses showed that the overall structures of Kaiso in complex with the KBS or methylated DNA are nearly identical and manifest similar modes of DNA recognition [26]. Kaiso binding to methylated DNA motifs is similar to that of its close relative ZBTB4 that binds mostly on one strand, and when the C^m^GC^m^G motif is bound, this could occur by two molecules of Kaiso, each on one DNA strand [18]. When DNA is replicated, it is hemi-methylated. Binding of Kaiso to the (still) methylated DNA strand could thus ensure maintenance of the methylation mark. This leaves the possibility that although the KBS in ICR1-B4 is essential, binding of Kaiso to the methylated sites in the ICR1 is also necessary for a proper ICR1 methylation maintenance. A loss of the KBS in ICR1-B4 is observed in BWS patients with ICR1 microdeletions that individually vary in size, ranging from 1.4 to 2.2 kbp and fusing the B-elements of the first cluster (B1-3) to the B-elements of the second cluster of ICR1 repeats (B5–B6) [6, 27, 28]. These deletions are on the maternal, normally unmethylated ICR1 allele and mostly affect CTS spacing. A CTS spacing similar to the intact ICR1 results in moderate ICR1 inactivation and is associated with stochastic gain of DNA methylation of the maternal ICR1, whereas microdeletions with altered CTS spacing result in severe ICR1 inactivation, associated with ICR1 hypermethylation [28]. CTS spacing thus seems to be essential for maintaining the unmethylated state of the maternal ICR1. If this spacing is disturbed, a de novo methylation can occur. Using CRISPR/Cas9, we generated a set of small deletions which ablated Kaiso binding to one or both alleles. Yet the DNA methylation status of the ICR1 is not increased, unlike what is observed in patients with the described ICR1-microdeletions, but rather a decrease was observed. This suggests that the deletions generated by CRISPR/Cas9 do not sufficiently perturb the spacing of factors bound to the ICR1 on the maternal allele. The decrease of methylation must occur on the paternal allele, and there is a causal connection of the epigenetic alteration with the hampering of Kaiso binding to the KBS. Nonetheless, we cannot exclude the possibility that Kaiso binding still has to occur in a certain pattern on the paternal ICR1 allele and not only to the KBS in B4 to maintain the methylation. The depletion of Kaiso or the deletion of the KBS in B4 is unlikely to affect CTCF binding to the ICR1, since we already could show that maternally transmitted ICR1 microdeletions mostly affect IC1 function and CTCF binding by changing CTCF binding site spacing. So does a 2.2-kb deletion that fuses B1 to B5 have an efficiency of CTCF binding similar to a wild-type ICR1, whereas a 1.8-kb deletion that fuses B2 to B5 has a lower CTCF binding efficiency, although in both cases, the complete B4 element with the KBS is deleted [28].

The observed demethylation of the ICR1 after Kaiso depletion by lentiviral knockdown as well as the CRISPR/Cas9 editing of the KBS in B4 goes along with a decrease of *IGF2* as well as of *H19* expression. How can Kaiso potentially mediate these transcriptional effects? From our current understanding of an unmethylated ICR1 being a prerequisite for *H19* transcription and *IGF2* inactivation, a hypomethylation of an intact ICR1 should result—as observed—in an *IGF2* decrease; however, it should not decrease also the transcription of *H19*. One explanation is that Kaiso binds indeed to the KBS in B4 on both parental alleles, since the binding motif cannot be methylated, but on the maternal allele, this binding exerts functions that strengthen the *H19* promoter and thus increase *H19* transcription. Support for this functional model comes from functional observations of a consensus KBS in close proximity to the CTS in the human 5′ beta-globin insulator. The presence of this KBS reduced the enhancer blocking activity of CTCF in an insulation assay, suggesting that the Kaiso-CTCF interaction negatively regulates CTCF insulator activity [29]. By comparison of the expression levels of genes whose proximal region including the promoter is bound by Kaiso in several cell lines versus the expression of the genes whose promoters are bound by Kaiso in a cell type-specific manner, a positive correlation between Kaiso binding and gene expression can be observed, suggesting that Kaiso may recruit positively acting transcription factors [30]. Although it is unlikely that Kaiso binding to the KBS affects CTCF binding efficiency [28], a composite CTCF- and Kaiso-KBS binding to the unmethylated ICR1 might exert a role in insulation, enhancer blocking and loop formation. Thus, Kaiso binding to the unmethylated ICR1 that is loaded with CTCF could suggestively also represent a regulatory mechanism for specific ICR1 function.

Since the KBS motif can be found also in other ICRs than the ICR1 (compare Additional file 2: Table S1), it can be speculated that Kaiso might have a methylation maintenance effect also on these. However, our preliminary data suggests that on at least four selected ICRs with a KBS, no methylation change can be observed after shRNA-mediated Kaiso depletion (compare Additional file 2: Table S1). In addition to the methylation, Kaiso binding in the ICR1 affects also the transcription of *IGF2* and *H19* and we discussed already that this might not directly be connected to the role of Kaiso in the methylation maintenance. From ten analysed imprinted genes, only three showed a transcriptional dysregulation after Kaiso knockdown, one being the already discussed *IGF2* and the others the *KCNQ1OT1* transcript and the *PEG3* gene (compare Additional file 2: Table S1). In the light of the proposed network of imprinted genes, it remains to be seen if the observed transcriptional effects might be mediated by the transcriptional finetuning of *H19*, that is known to act as a transfactor for transcriptional regulation of other imprinted genes [31].

What are the physiological consequences of Kaiso binding to the ICR1? It is known that Kaiso regulates several genes including *MMP7* [32, 33] and β-catenin [34]. However, deletion of the Kaiso gene causes no overt phenotype in mice. Yet Kaiso-deficient *Apc*^Min/+^ mice, a model for human familial adenomatous polyposis [35], exhibit delayed intestinal tumourigenesis [36]. In this context, it is also noteworthy that Kaiso DNA binding and transcriptional activity is attenuated by the interaction with the p120 catenin protein [37], which is commonly reduced or lost in colorectal tumours [38]. *H19* has been observed to be transcriptionally upregulated in a number of tumours, and this upregulation is correlated with the increased metastatic potential of several tumours [39]. If Kaiso is responsible for *H19* transcriptional fine tuning, *H19* could be downregulated in the case of Kaiso binding loss to the ICR1 and thus explain the protective findings in Kaiso-deficient *Apc*^Min/+^ mice [36]. Furthermore, Nakagawa and coworkers demonstrated that methylation-dependent loss of *IGF2* imprinting was present in colon tumours and normal colonic mucosa, suggesting that *IGF2* overexpression predisposes to cancer [40]. If Kaiso binding to the ICR1 is necessary for ICR1 methylation maintenance and *IGF2* expression from the methylated alleles, Kaiso-deficient Apc^Min/+^ mice should display reduced ICR1 methylation and consequently also reduced *Igf2* expression (as suggested by our knockdown experiments, see Fig. 3), also explaining the delayed intestinal tumourigenesis. Thus, the concerted reduction of *H19* and *IGF2* due to reduced Kaiso binding to the ICR1 makes Kaiso an interesting potential target for therapeutic *interventions in cases of disease assoc*iation with *H19*/*IGF2* overexpression.

## Conclusions

Our studies demonstrate that Kaiso (ZBTB33) binds to its unmethylated recognition site within the human ICR1 and that this binding is essential for the methylation maintenance of the paternal ICR1 allele and affects the transcriptional regulation of the *H19* gene. The methylation of ICR2 on the same chromosome was unaffected by the Kaiso depletion or ICR1-KBS deletion, supporting the idea that Kaiso is an essential factor particular for ICR1 function and represents the long-sought alternative DMR methylation maintenance factor for ICR1. Further studies are required to identify the mechanisms responsible for these Kaiso-mediated effects. Yet our findings open up new mechanistic insights into the basic ICR1 functional regulation with implications on imprinting diseases like BWS/SRS as well as on different tumour entities.

## Methods

### Cell culture

Human primary fibroblasts with confirmed differential methylation of the ICR1 were obtained with informed written consent and ethics approval by the Independent Ethics Committee (EK 159/08, Aachen). The study was conducted according to the ethical principles of the Declaration of Helsinki. Phoenix cells (HEK293T) (obtained from JP) and primary fibroblast cells were grown in DMEM (Gibco) and RPMI (Gibco), respectively, supplemented with 10 % FCS, 1 mM sodium pyruvate, 100 U/ml penicillin and 100 μg/ml streptomycin.

### Electrophoretic mobility shift assay (EMSA)

Single-stranded 5′-fluorescence-labelled (IRDyeTM-700) forward and reverse oligonucleotides containing the regular entire ICR1-KBS (5′-AGGGTCTCTGGCAGGCACAGAGCC-3′), B4-KBS (5′-GGCCTTTGGCAGGAACAGG-3′) or the known MMP7-KBS (5′-GTGCTTCCTGCCAATAACG-3′) were annealed. The full-length Kaiso-ORF was cloned into a pT7-CFE1-NFtag vector (Pierce) and in vitro translated by the use of modified HeLa cell extracts to generate functional full-length N-terminally Flag-tagged protein according to the manufacturer’s protocol (Pierce). In supershift reactions, Flag-tagged Kaiso was incubated with anti-Kaiso (Abcam), anti-Flag antibody (Sigma-Aldrich) or unspecific IgG antibodies (Diagenode) blocked by BSA. Protein extracts of in vitro translated pT7-CFE1-NFtag vector backbone were used as additional negative controls. In competition experiments, the protein was incubated with unlabelled competitor oligos. All reactions were analysed on a 4 % TBE polyacrylamide gel containing 2.5 % glycerol and scanned with the Odyssey imager (LI-COR Biosciences).

### ChIP assay

ChIP was performed using a modified protocol of Nelson, Denisenko and Bomsztyk (37) and the SimpleChIP kit protocol (NEB). Primary fibroblasts (1 × 10^7^) of a proband with normal differentially methylated ICR1 were cross-linked with a total of 1 % fresh formaldehyde (Thermo) and harvested as described [41]. Chromatin was fragmented in 90 cycles with a HTU-Soni130-CV188 sonicator. Chromatin samples were incubated with anti-Kaiso antibody (Bethyl) or unspecific IgG antibodies (Diagenode) and bound to magnetic beads for 2× washing with IP buffer, low and high salt washes (NEB). Elution of chromatin from beads and reversal of cross-links were done according to the SimpleChIP kit protocol (NEB). Analysis of pulldown efficiency was performed by qPCR using primers amplifying complete B-repeats B1-3, B5-6 (5′-GGGCTCTTGCRTAGCACATG-3′, 5′-TGTGATGTGKGAGCCTGCAC-3′), B4 (5′-AGGTGATCATGACTGGGACC-3′, 5′-AGACTCCAGGAACACTGTGC-3′) and *MMP7* as previously published [41].

### Lentiviral shRNA transfections

Downregulation of Kaiso in human primary fibroblasts was achieved by infecting primary human fibroblasts with human Kaiso-specific shRNAs in lentiviral particles (SC38019-V) according to the manufacturer’s recommendations (Santa Cruz Biotechnology, Inc.). Scrambled shRNA with no endogenous mRNA target (SC108080) was used as control (Santa Cruz Biotechnology, Inc.). At 24 h post infection, cells were incubated in RPMI with 10 % FCS (Gibco) and selected for puromycin resistance due to the integration and active transcription of the shRNA expression construct.

### CRISPR/Cas9-based genome editing

Two different synthetic guide RNAs (sgRNA) targeting the human KBS-B4 sequence were synthesized as corresponding double-stranded DNA oligos and after annealing cloned into the *HpaI-XhoI* sites downstream of the human U6 promoter in the engineered lentiviral plasmid pLeGO [23] as previously published [41]. A sgRNA targeting the murine Rosa26 locus was used as a negative control. Retroviral packaging was performed using ecotropic Phoenix cells (HEK293T). Lentivirus production in 293T/17 (ATCC) cells was performed by calcium phosphate transfection as previously described [42]. Lentiviral supernatant was collected 48, 56, 64 and 72 h post-transfection, pooled and precipitated with PEG6000 and 0.3 M NaCl. Aliquots (100 μl) mixed with an equal volume of media were incubated with human primary fibroblast target cells for approximately 7 days, and GFP^+^ cells were sorted by flow cytometry. On average, 1–5 % of cells were positive for the uptake/integration of the genome editing constructs.

### RNA purification, cDNA synthesis and quantitative PCR

RNA extraction was performed using the peqGOLD total RNA kit (PeqLab) according to the manufacturer’s protocol. cDNA synthesis with oligo-dT priming was performed using the M-MLV reverse transcriptase from Invitrogen. For qPCR, the KAPA SYBR FAST Master Mix (PeqLab) and LightCycler 480 detection system (Roche) were used. *PDH* and *B2M* expressions were measured as controls for sample normalization.

### Bisulfite pyrosequencing

Genomic DNA of primary fibroblasts was extracted with the High Pure PCR Template preparation Kit (Roche) according to the manufacturer’s instructions. Bisulfite conversion of 500 ng genomic DNA per sample was performed with the EpiTect Bisulfite Kit (Qiagen) according to the manufacturer’s specifications. Quantification of DNA methylation was carried out by PCR of bisulfite-converted DNA and pyrosequencing using a PyroMark Q96 ID instrument (Qiagen) and PyroMark Gold Q96 reagents (Qiagen). Data were analysed using the PyroMark CpG Software 1.0.11 (Qiagen).

### Statistics

An independent two-sided *t* test was carried out for the statistical analysis in the qPCR experiments following shRNA-based knockdown or sgRNA-based genomic editing in compared cell populations. Significance of the paired ChIP experiments was calculated with a paired two-sided *t* test. Values from all experiments were used for calculation of the means and the respective SEMs.

## Additional files

Additional file 1: Figure S1. Reduced binding of Kaiso to the ICR1 does not alter the methylation of the KvDMR1/ICR2. ICR1 methylation was determined by pyrosequencing of two established differentially methylated KvDMR1/ICR2 CpGs in bisulfite-treated DNA. Displayed histogram values are represented as mean ± SEM from two replicate analyses for each CG position. (A) Knockdown of Kaiso is not associated with reduced methylation of endogenous KvDMR1/ICR2. (B) Cas9-modified fibroblasts impaired for ICR1 methylation due to ICR1-BS editing are not impaired for KvDMR1/ICR2 methylation. (JPG 129 kb) Additional file 2: Table S1. Influence of Kaiso on other ICRs. Other ICRs than the ICR1 based on (Court et al. [43]) on different chromosomes were analysed for the presence of KBS and their number listed. In addition, the presence of ZPF57 target sites (ZFP57TS) was analysed (Y = yes, N = no) as well as the Kaiso knockdown effect on the methylation of the DMR and the transcription of flanking imprinted genes of the respective ICRs (n.d. = not determined, NO = no change observed, UP = upregulated, DOWN = downregulated). (XLSX 13 kb)

## Abbreviations

BWS: Beckwith-Wiedemann syndrome
SRS: Silver-Russell syndrome
ICR1: imprinting control region 1
ICR2: imprinting control region 2
KBS: unmethylated Kaiso binding site
