# Supplementary figures and images for: Kaiso mediates human ICR1 methylation maintenance and H19 transcriptional fine regulation
Source: Clin Epigenetics. 2016 May 4;8:47. doi: 10.1186/s13148-016-0215-4 (PMC4857248; doi:10.1186/s13148-016-0215-4)

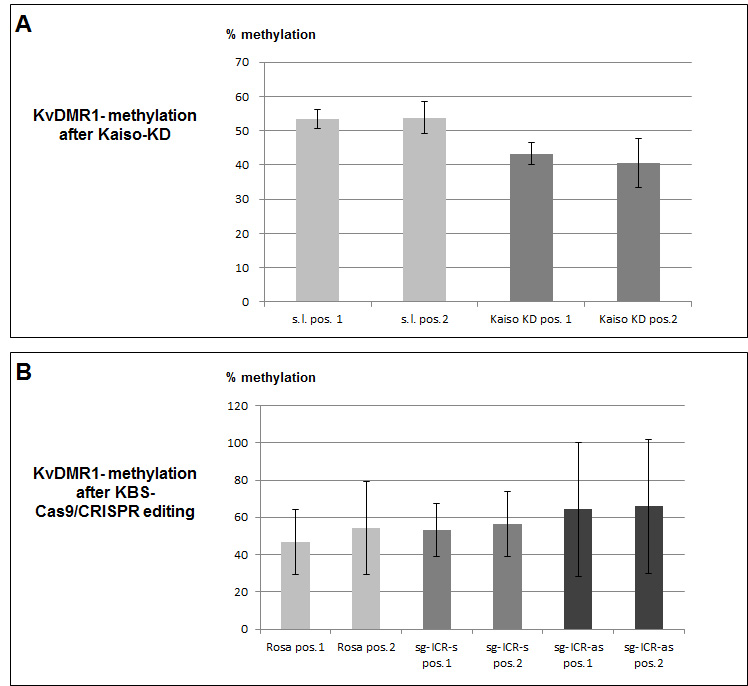

Supplement: Additional file 1: Figure S1. — Reduced binding of Kaiso to the ICR1 does not alter the methylation of the KvDMR1/ICR2. ICR1 methylation was determined by pyrosequencing of two established differentially methylated KvDMR1/ICR2 CpGs in bisulfite-treated DNA. Displayed histogram values are represented as mean ± SEM from two replicate analyses for each CG position. (A) Knockdown of Kaiso is not associated with reduced methylation of endogenous KvDMR1/ICR2. (B) Cas9-modified fibroblasts impaired for ICR1 methylation due to ICR1-BS editing are not impaired for KvDMR1/ICR2 methylation. (JPG 129 kb) [file 13148_2016_215_MOESM1_ESM.jpg]
